# Supplementary material for: Neural crest cell-derived DKK1 and NEDD4 modulate Wnt signalling in the second heart field to orchestrate outflow tract development
Source: Nat Commun. 2026 Jan 22;17:1751. doi: 10.1038/s41467-026-68459-4 (PMC12913648; doi:10.1038/s41467-026-68459-4)
Supplement: Supplementary file 1 — Supplementary Information [file 41467_2026_68459_MOESM1_ESM.pdf]

Supplementary Information:

Neural crest cell-derived DKK1 and NEDD4 modulate Wnt signalling in the second heart field to orchestrate outflow tract development

Supplementary Figure 1

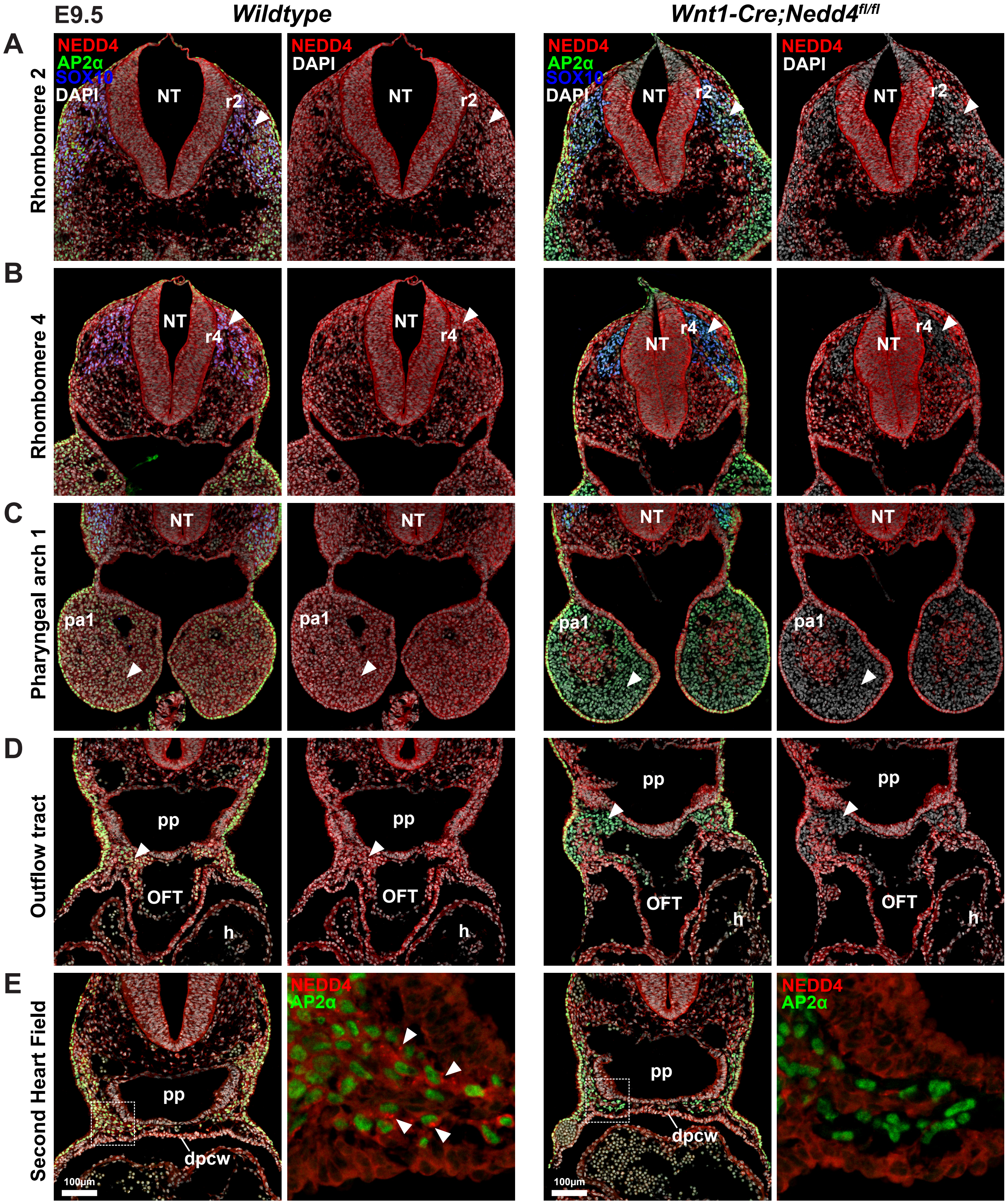

Supplementary Figure 1 – NEDD4 is broadly expressed throughout embryonic tissues including neural crest cells.

Transverse sections of E9.5 *wildtype* and *Wnt1-Cre; Nedd4*$^{fl/fl}$ embryos immunostained for NEDD4, AP2α (pan-neural crest cell marker) and SOX10 (early migrating neural crest cell marker). NEDD4 is broadly expressed throughout all tissues shown in **A:** Rhombomere 2, **B:** Rhombomere 4, **C:** Pharyngeal arch 1, **D:** Outflow tract, **E:** Second heart field. Arrowheads indicate AP2α-positive neural crest cells, which show specific loss of NEDD4 expression in *Wnt1-Cre; Nedd4*$^{fl/fl}$ embryos as expected. Arrowheads in (**E**) highlight enhanced and punctate expression of NEDD4 specifically in neural crest cells that are located near the second heart field region. Representative images from n=4 independent experiments.

Supplementary Figure 2

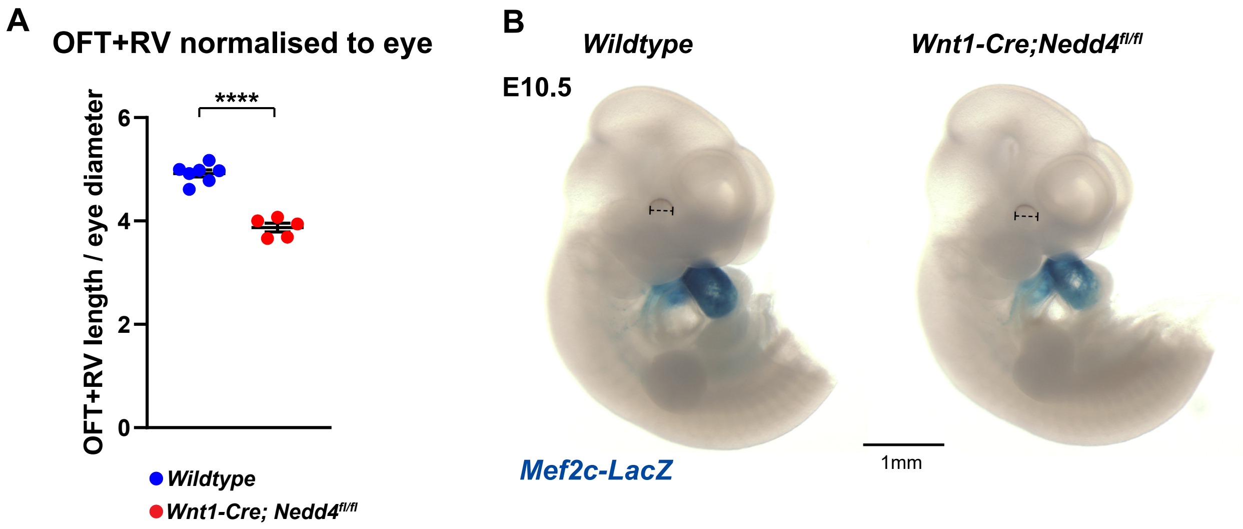

Supplementary Figure 2 – Outflow tract is shortened in *Wnt1-Cre; Nedd4$^{fl/fl}$* embryos at E10.5, while whole embryo growth is unaffected.

A: Outflow tract and right ventricle length was measured at E10.5 and expressed graphically normalised to the eye diameter of each embryo, shown by the dashed lines in (**B**), representing an unaffected structure. Data points represent biological replicates from n=7 *wildtype* and n=5 *Wnt1-Cre; Nedd4$^{fl/fl}$* embryos from 3 independent experiments. Mean +/- SEM, ****p=0.000002, unpaired two-tailed t-test.

B: E10.5 *wildtype* and *Wnt1-Cre; Nedd4$^{fl/fl}$* embryos stained with X-gal and highlighting the outflow tract and right ventricle by expression of *Mef2c-LacZ*. Gross embryo growth, size and morphology are equivalent between genotypes. Source data are provided as a Source Data file.

Supplementary Figure 3

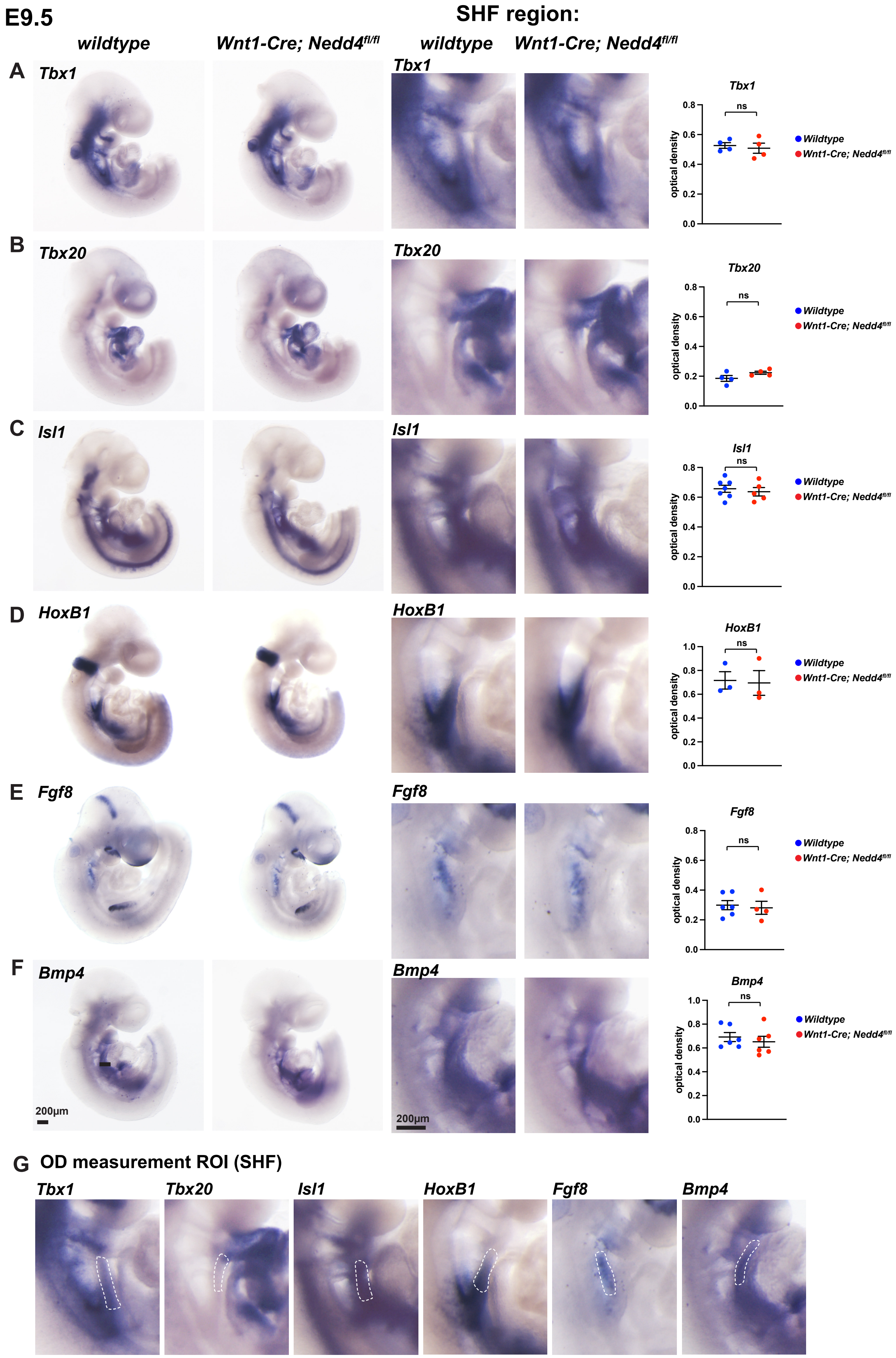

Supplementary Figure 3 – Heart field and cardiac development markers appear unchanged in *Wnt1-Cre; Nedd4$^{fl/fl}$* embryos.

Whole mount *in situ* hybridisation on E9.5 *wildtype* and *Wnt1-Cre; Nedd4$^{fl/fl}$* embryos for **A:** *Tbx1*, **B:** *Tbx20*, **C:** *Isl1*, **D:** *HoxB1*, **E:** *Fgf8*, and **F:** *Bmp4*. *In situ* hybridisation was performed on a minimum of 3 and up to 7 embryos per probe per genotype. The second heart field (SHF) region which is of interest for this study is shown at higher magnification in the inset images, and appears unchanged between genotypes. Optical density of BCIP/NBT substrate staining was measured in the SHF, with the region of interest measured shown by the dashed line in examples in **G**. This quantification is presented graphically for each probe, with individual data points representing values for independent whole embryos. Mean +/- SEM; ns, not significant; unpaired two-tailed t-test. Source data are provided as a Source Data file.

Supplementary Figure 4

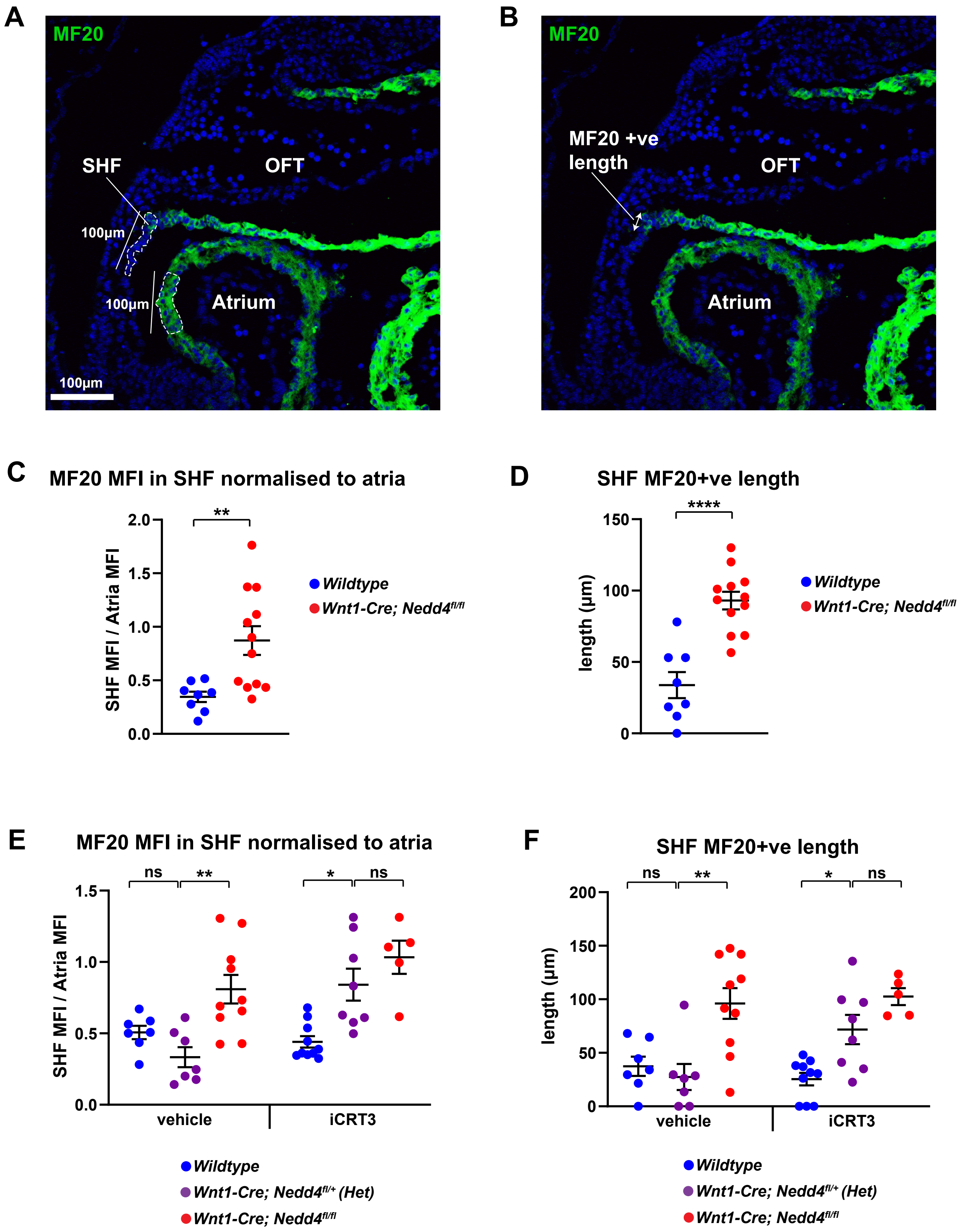

Supplementary Figure 4 – Additional quantitation methods for MF20 immunostaining in the anterior second heart field.

A: Example of the method for defining the region of interest used for MF20 mean fluorescence intensity (MFI) measurements. A line 100μm in length was drawn at an angle perpendicular to the OFT and parallel to the pericardial wall. This was used as a guide to determine the extent of the area of interest of the anterior second heart field (SHF), which was traced (shown as dashed line) to define the region of interest for MFI measurements for graphs shown in Fig. 3G and Fig. 5F. Additionally, the MF20 MFI in an area unaffected by NEDD4 removal in the neural crest was measured as an internal normalisation control, and is indicated by the dashed line surrounding a 100μm long region in the wall of the atria. **B:** Example of the method used for defining the length of MF20 +ve immunostaining in the anterior SHF. The dashed line with arrowheads indicates the length of MF20 +ve staining, as measured beginning from the junction of the OFT with the pericardial wall. **C:** MF20 MFI measurements as in Fig. 3G that have been normalised to atria MFI within the same acquired images. Mean +/- SEM; **p=0.0065. Unpaired two-tailed t-test. **D:** MF20 +ve length measurements for all samples represented in Fig. 3G. Mean +/- SEM; ****p=0.000028. Unpaired two-tailed t-test. **E:** MF20 MFI measurements as in Fig. 5F that have been normalised to atria MFI within the same acquired images. Mean +/- SEM; ns, not significant; **p=0.0027; *p=0.0118. 2 way ANOVA multiple comparisons. **F:** MF20 +ve length measurements for all samples represented in Fig. 5F. Mean +/- SEM; ns, not significant; **p=0.0013; *p=0.045. 2 way ANOVA multiple comparisons. Source data are provided as a Source Data file.

Supplementary Figure 5

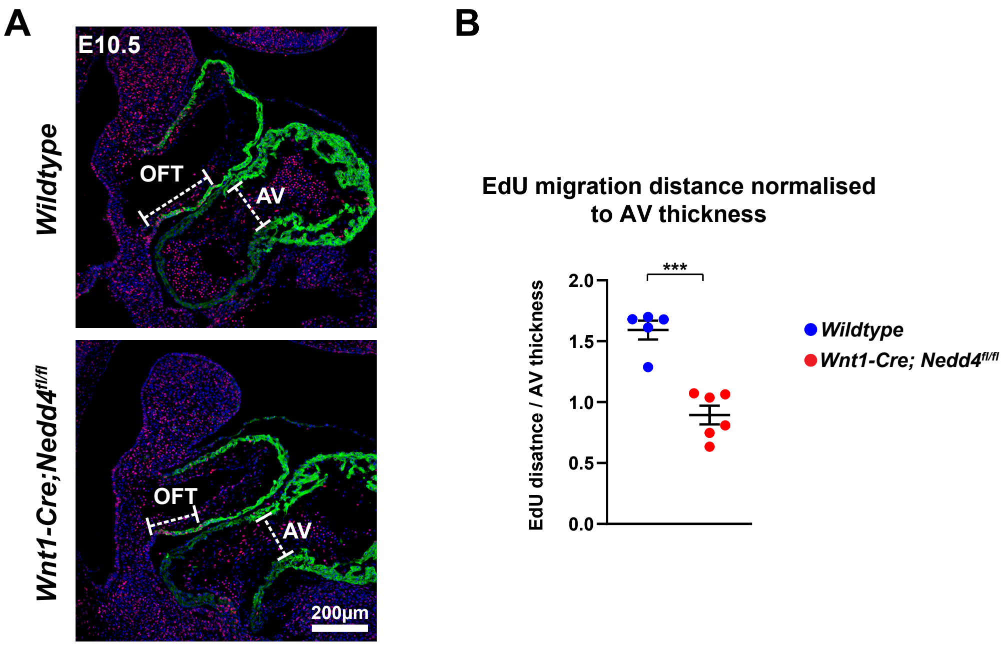

Supplementary Figure 5 - Additional quantitation method for EdU outflow tract deployment assay.

A: Example of method used to normalise EdU migration front distance. The atrioventricular valve (AV) primordium thickness (indicated by dashed lines) is a structure that should not be affected by loss of NEDD4 in neural crest cells. This is present in the same acquired images used for EdU migration front distance measurements in the outflow tract (OFT). **B:** EdU migration front distance measurements as in Fig. 3J were normalised to AV thickness measurements to account for variations in embryo size. Data points represent biological replicates from n=6 *wildtype* and n=6 *Wnt1-Cre; Nedd4*$^{fl/fl}$ embryos from 3 independent experiments. Mean +/- SEM; ***p=0.0001. Unpaired two-tailed t-test. Source data are provided as a Source Data file.

Supplementary Figure 6

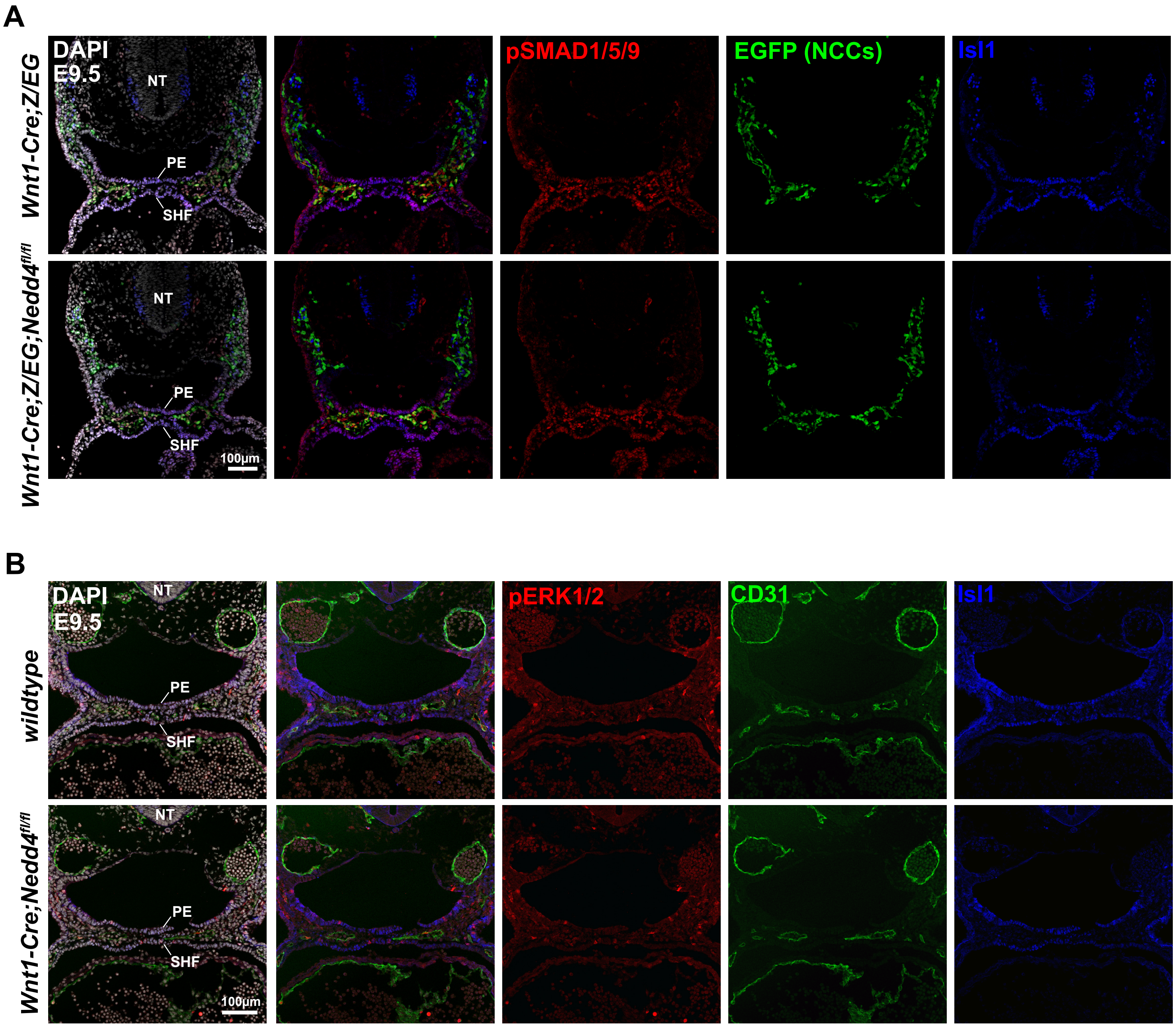

Supplementary Figure 6 – Phospho-SMAD and Phospho-ERK signalling appears unchanged in the second heart field region of *Wnt1-Cre; Nedd4$^{fl/fl}$* embryos.

A: Transverse sections of E9.5 *Wnt1-Cre; Z/EG* and *Wnt1-Cre; Z/EG; Nedd4$^{fl/fl}$* embryos immunostained for phospho-SMAD1/5/9, EGFP and Isl1. **B:** Transverse sections of E9.5 *wildtype* and *Wnt1-Cre; Nedd4$^{fl/fl}$* embryos immunostained for phospho-ERK1/2, CD31 and Isl1. NT, neural tube; PE, pharyngeal endoderm; SHF, second heart field.

Supplementary Figure 7

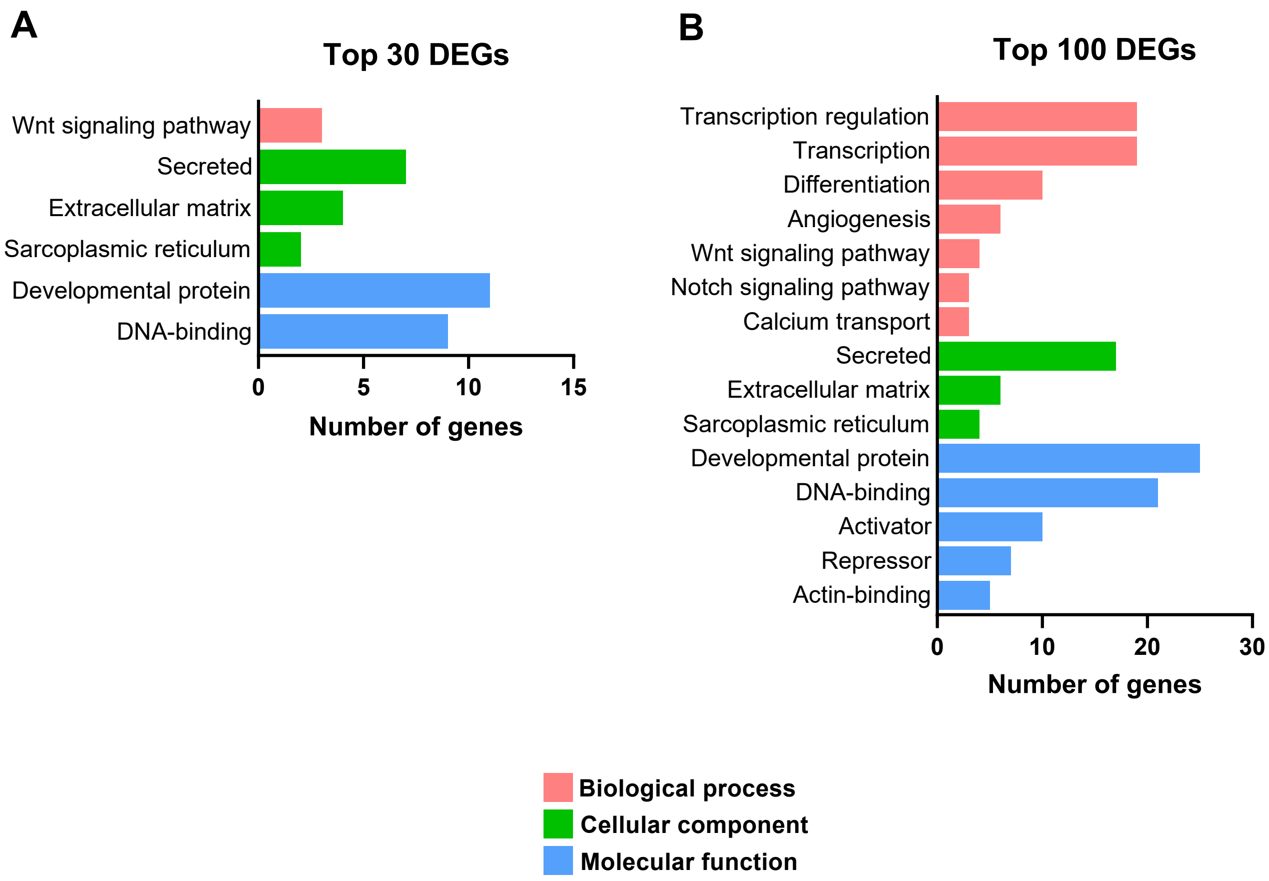

Supplementary Figure 7 – Gene ontology analysis of differentially expressed genes in *Wnt1-Cre; Nedd4$^{fl/fl}$* compared to *wildtype* embryos.

A: Gene ontology analysis (DAVID) of the top 30 differentially expressed genes (DEGs) from laser capture RNA-seq analysis of the second heart field region from *wildtype* and *Wnt1-Cre;Nedd4$^{fl/fl}$* embryos. Terms assessed were "Biological process", "Cellular component" and "Molecular function". **B:** Gene ontology analysis of the top 100 DEGs, as in A.

Supplementary Figure 8

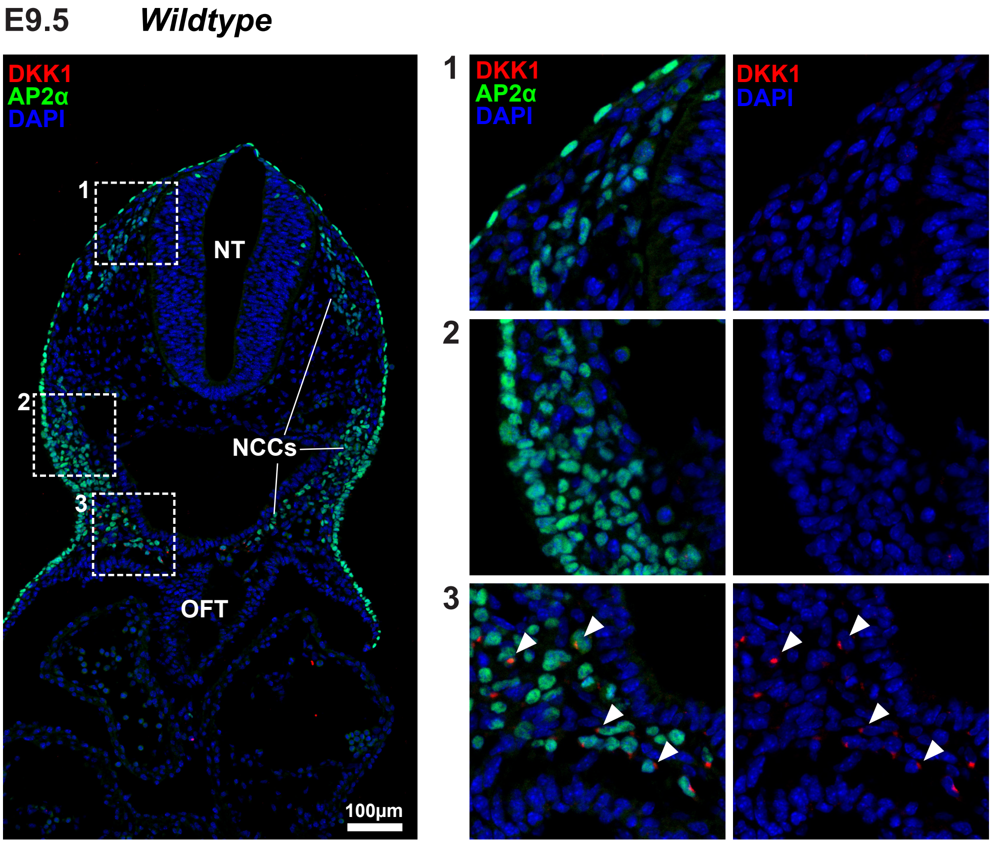

Supplementary Figure 8 – DKK1 is specifically expressed in a subset of neural crest cells that are closely associated with the second heart field.

Transverse section of a *wildtype* E9.5 embryo through the outflow tract region, immunostained for DKK1 and AP2α. Higher magnification images are shown corresponding to the numbered dashed boxes. 1: Early migrating post-delamination neural crest cells, 2: Mid-migrating neural crest cells, 3: Later migrating neural crest cells that have reached the second heart field region. DKK1 immunostaining is specific to neural crest cells as indicated by overlapping DKK1 and AP2α. Importantly, DKK1 expression is restricted only to neural crest cells which have arrived at the second heart field region, and is absent in early migrating neural crest cells, suggesting a critical temporal role for DKK1 specifically in cardiac neural crest cells. Representative images from n=6 independent experiments.

Supplementary Figure 9

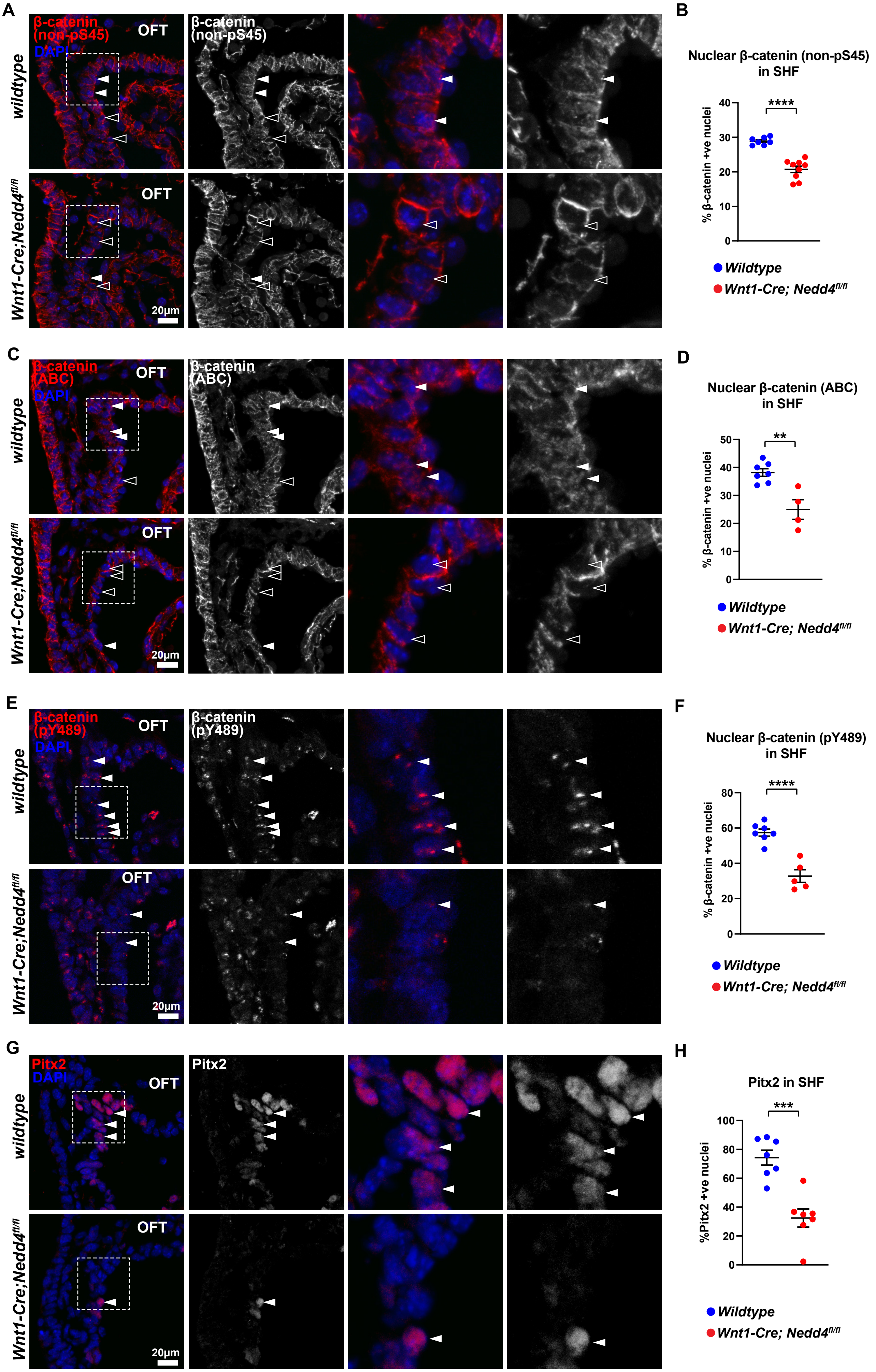

Supplementary Figure 9 – Canonical Wnt signalling is downregulated in the second heart field of *Wnt1-Cre; Nedd4$^{fl/fl}$* embryos.

A: Sagittal sections through the outflow tract region of E9.5 *wildtype* and *Wnt1-Cre; Nedd4$^{fl/fl}$* embryos immunostained for β-catenin non-phospho serine 45 (as in Fig. 4H). Higher magnification image is shown of the dashed box area. Solid arrowheads point to examples of β-catenin +ve nuclei. Outlined arrowheads point to examples of cells with no nuclear localised β-catenin. **B:** Quantification of β-catenin +ve nuclei (as in Fig. 4H) from immunostaining shown in (A). Mean +/- SEM; ****p=0.000005. Unpaired two-tailed t-test. **C:** Immunostaining of E9.5 *wildtype* and *Wnt1-Cre; Nedd4$^{fl/fl}$* embryos for Active β-catenin (ABC). Solid arrowheads point to examples of β-catenin +ve nuclei. Outlined arrowheads point to examples of cells with no nuclear localised β-catenin. **D:** Quantification of β-catenin (ABC) +ve nuclei from immunostaining shown in (C). Mean +/- SEM; **p=0.0022. Unpaired two-tailed t-test. **E:** Immunostaining of E9.5 *wildtype* and *Wnt1-Cre; Nedd4$^{fl/fl}$* embryos for β-catenin phospho-tyrosine 489. Solid arrowheads point to examples of β-catenin +ve nuclei. **F:** Quantification of β-catenin pY489 +ve nuclei from immunostaining shown in (E). Mean +/- SEM; ****p=0.000073. Unpaired two-tailed t-test. **G:** Immunostaining of E9.5 *wildtype* and *Wnt1-Cre; Nedd4$^{fl/fl}$* embryos for Pitx2. Solid arrowheads point to examples of Pitx2 +ve nuclei. **H:** Quantification of Pitx2 +ve nuclei from immunostaining shown in (G). Mean +/- SEM; ***p=0.0002. Unpaired two-tailed t-test. Source data are provided as a Source Data file.

Supplementary Figure 10

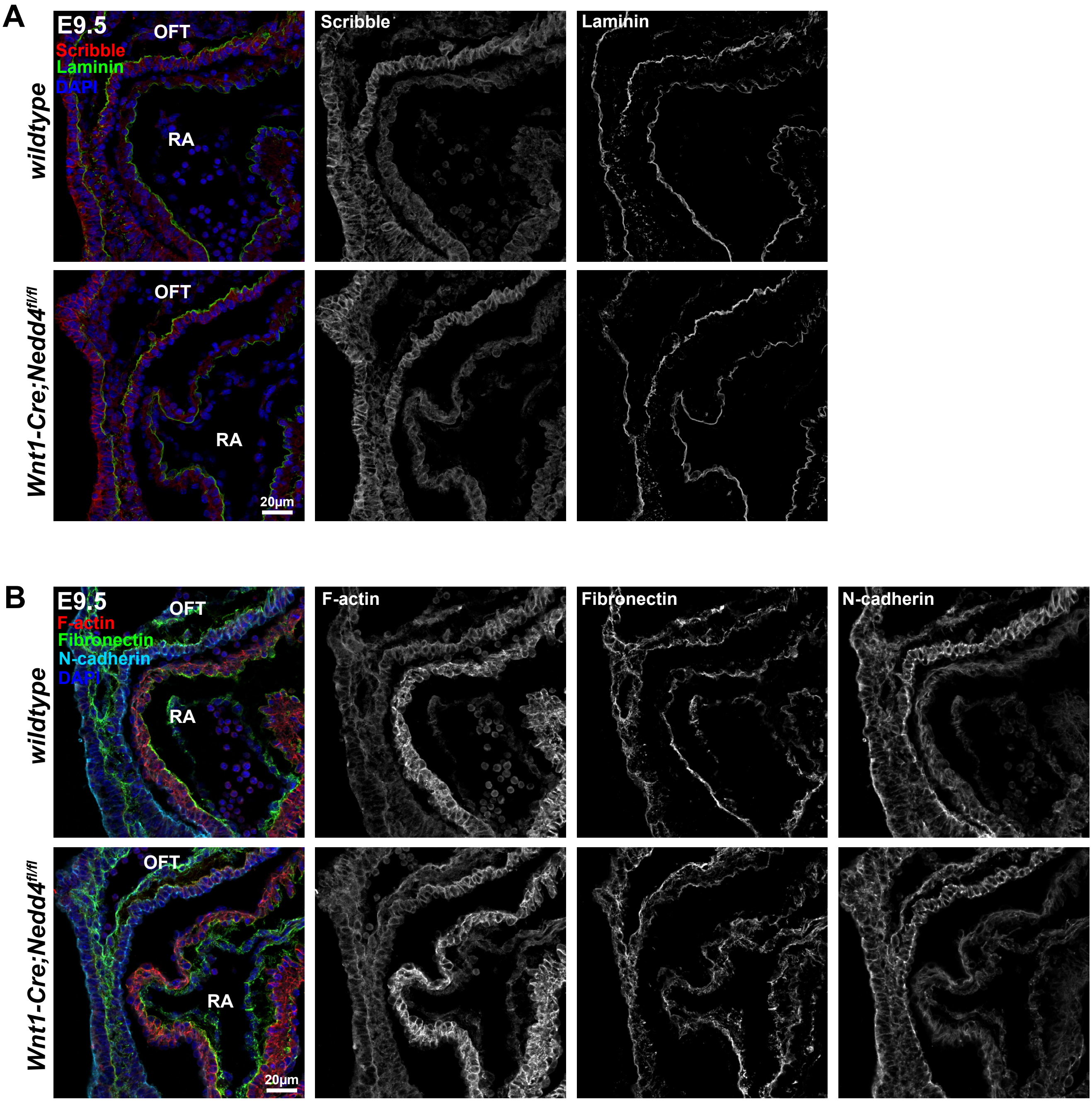

Supplementary Figure 10 – Cell polarity and extracellular matrix markers appear unchanged in the second heart field of *Wnt1-Cre; Nedd4$^{fl/fl}$* embryos.
A: Sagittal sections through the outflow tract region of E9.5 *wildtype* and *Wnt1-Cre; Nedd4$^{fl/fl}$* embryos immunostained for Scribble and Laminin. **B:** Sagittal sections through the outflow tract region of E9.5 *wildtype* and *Wnt1-Cre; Nedd4$^{fl/fl}$* embryos immunostained for F-actin with Phalloidin, Fibronectin and N-cadherin. OFT, outflow tract; RA, right atrium. Representative images from n=3 *wildtype* and n=3 *Wnt1-Cre; Nedd4$^{fl/fl}$* embryos from 3 independent experiments.

Supplementary Figure 11

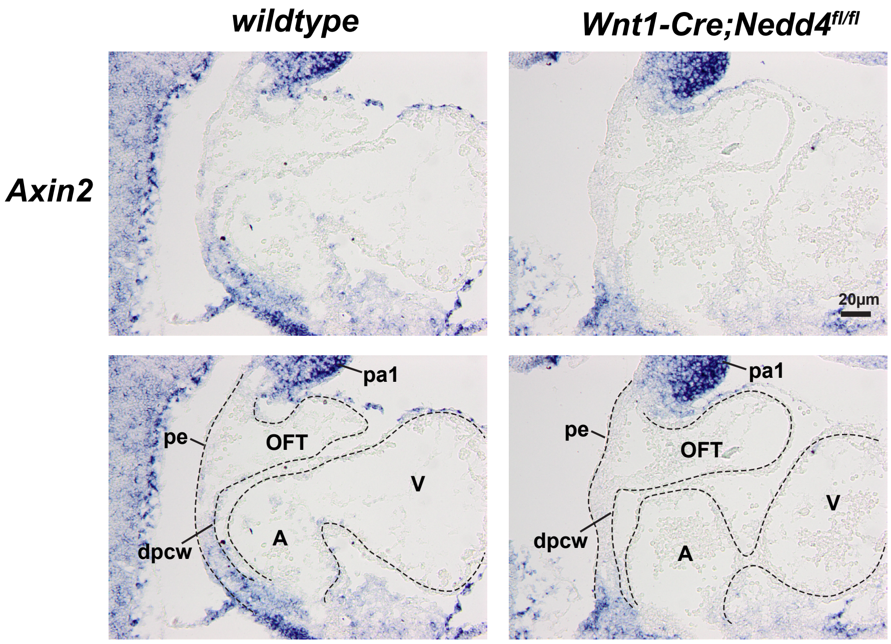

Supplementary Figure 11 – *Axin2 in situ* hybridisation

Uncropped images of *in situ* hybridisation for *Axin2* as shown in Figure 4K. Expression is evident in other areas such as the pharyngeal arch and posterior pharyngeal endoderm. Dashed lines in lower panels show overlay of tissue boundaries. pa1**,** pharyngeal arch 1; pe, pharyngeal endoderm; OFT, outflow tract; dpcw, dorsal pericardial wall; A, atrium; V, ventricle. Representative images from n=3 *wildtype* and n=3 *Wnt1-Cre; Nedd4*$^{fl/fl}$ embryos from 3 independent experiments.

Supplementary Figure 12

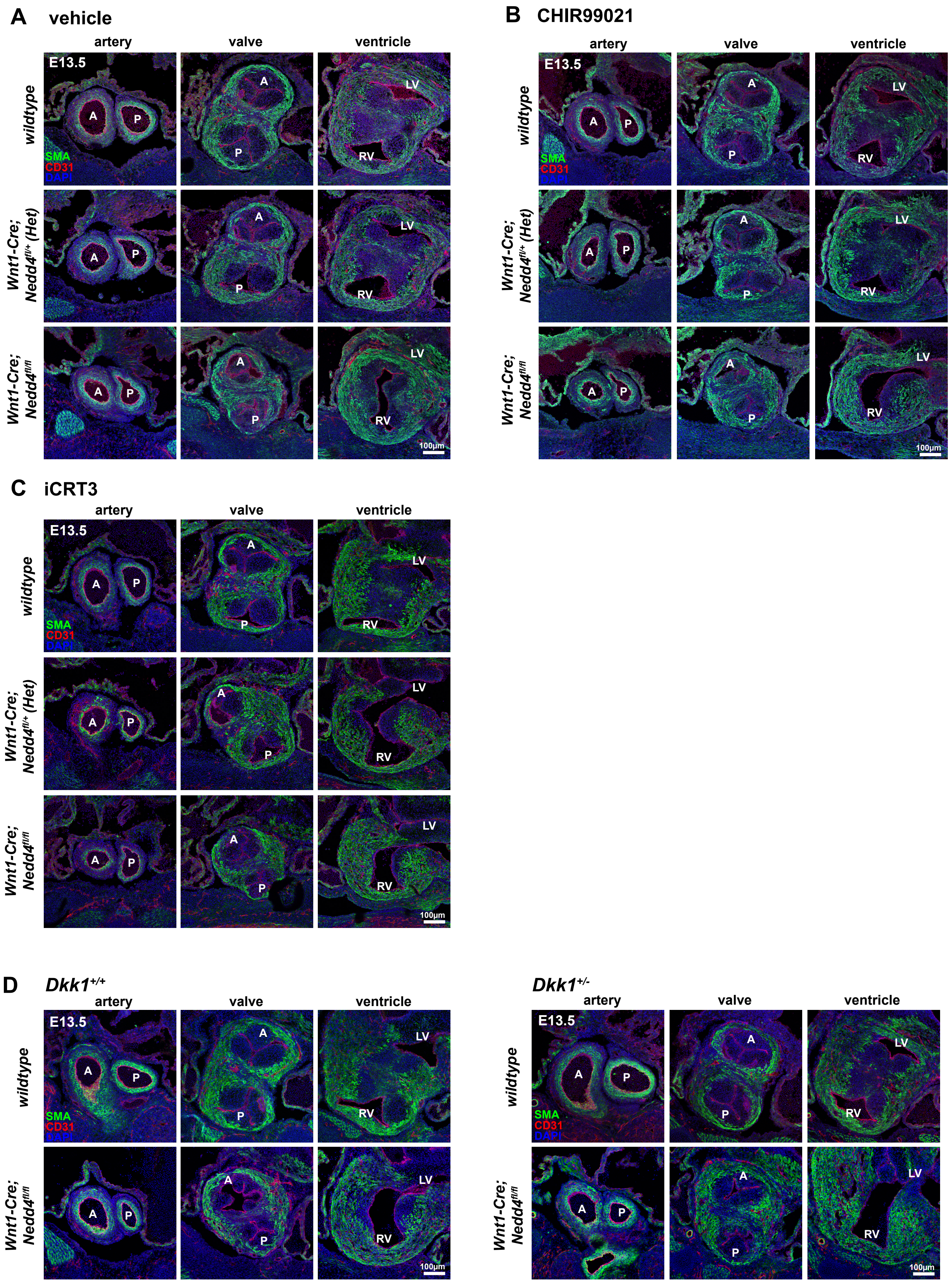

Supplementary Figure 12 – Outflow tract rotation at E13.5 in CHIR99021 and iCRT3 treated embryos, and with *Dkk1+/-* allele.

Sections through the outflow tract at multiple levels (artery, valve, ventricle), corresponding to the same embryo from the representative images shown in Fig. 5B and 5H. **A:** Coronal sections through the outflow tract region of vehicle treated *wildtype*, *Wnt1-Cre; Nedd4*$^{fl/+}$ and *Wnt1-Cre; Nedd4*$^{fl/fl}$ E13.5 embryos immunostained for smooth muscle actin (SMA) and CD31. **B:** Coronal sections through the outflow tract region of CHIR99021 treated *wildtype*, *Wnt1-Cre; Nedd4*$^{fl/+}$ and *Wnt1-Cre; Nedd4*$^{fl/fl}$ E13.5 embryos. **C:** Coronal sections through the outflow tract region of iCRT3 treated *wildtype*, *Wnt1-Cre; Nedd4*$^{fl/+}$ and *Wnt1-Cre; Nedd4*$^{fl/fl}$ E13.5 embryos. **D:** Coronal sections through the outflow tract region of *wildtype* and *Wnt1-Cre; Nedd4*$^{fl/fl}$ E13.5 embryos, either wildtype for *Dkk1* (*Dkk1*$^{+/+}$) or heterozygous for *Dkk1* (*Dkk1*$^{+/-}$). A, aortic artery/valve; P, pulmonary artery/valve; RV, right ventricle; LV, left ventricle.

Supplementary Figure 13

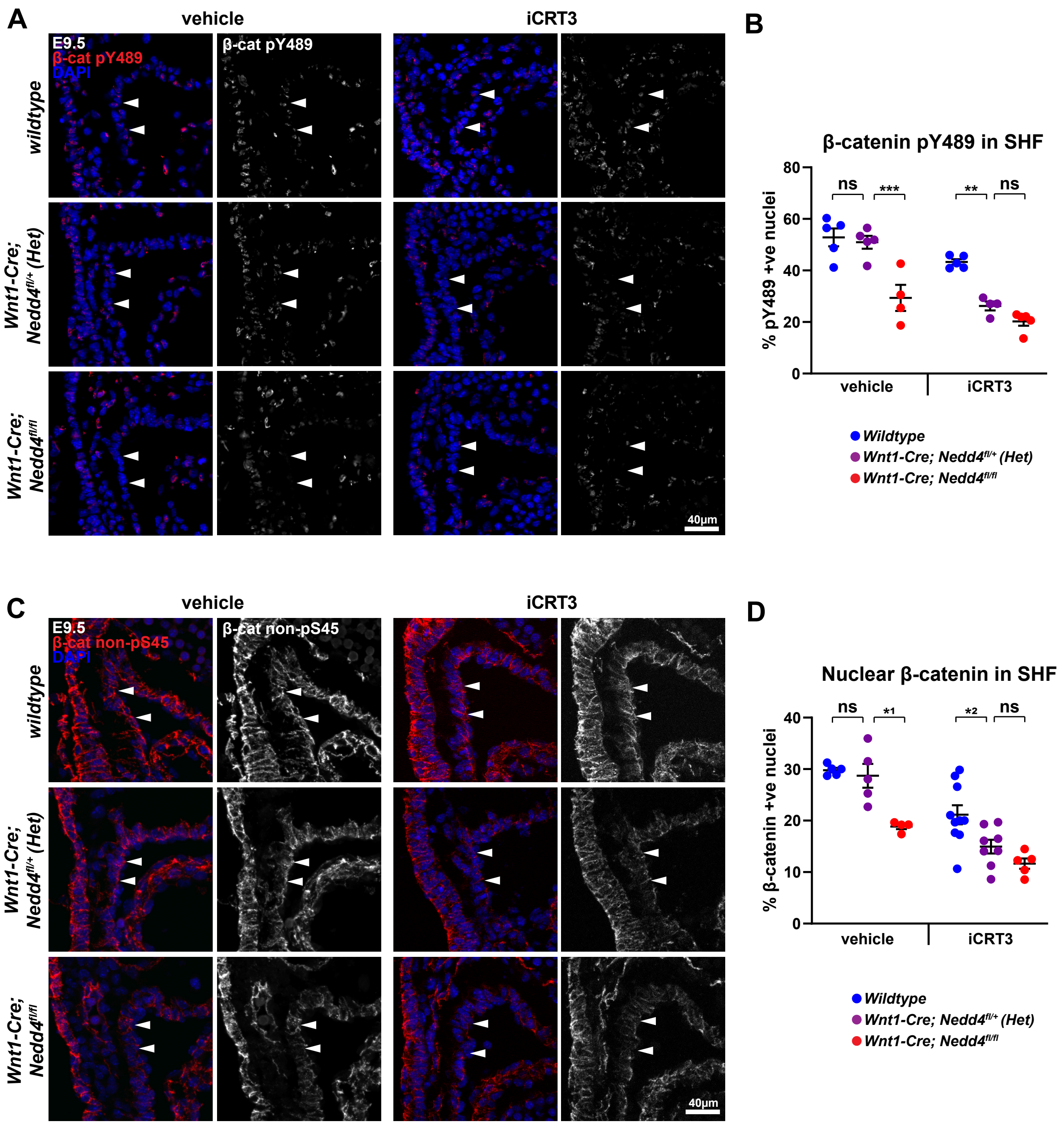

Supplementary Figure 13 – Canonical Wnt signalling activity in the second heart field of iCRT3 treated embryos at E9.5.

A: Sagittal sections through the outflow tract region of E9.5 vehicle and iCRT3 treated *wildtype*, *Wnt1-Cre; Nedd4$^{fl/+}$* and *Wnt1-Cre; Nedd4$^{fl/fl}$* embryos immunostained for β-catenin phospho-Y489. Arrowheads indicate the second heart field. **B:** Quantification of β-catenin pY489 +ve nuclei in the second heart field measured from immunostaining shown in (A). iCRT3 treatment reduced β-catenin pY489 +ve nuclei percentage in the SHF of *Wnt1-Cre; Nedd4$^{fl/+}$* (*Het*) embryos, to levels indistinguishable from *Wnt1-Cre; Nedd4$^{fl/fl}$* (*mutants*). Mean +/- SEM; ns; not significant; ***p=0.003; **p=0.0039, 2 way ANOVA Multiple Comparisons. **C:** Sagittal sections through the outflow tract region of E9.5 vehicle and iCRT3 treated *wildtype*, *Wnt1-Cre; Nedd4$^{fl/+}$* and *Wnt1-Cre; Nedd4$^{fl/fl}$* embryos immunostained for β-catenin non-phospho S45. Arrowheads indicate the second heart field. **D:** Quantification of β-catenin non-phospho S45 +ve nuclei in the second heart field measured from immunostaining shown in (C). iCRT3 treatment reduced β-catenin non-phospho S45 +ve nuclei percentage in the SHF of *Wnt1-Cre; Nedd4$^{fl/+}$* (*Het*) embryos, to levels indistinguishable from *Wnt1-Cre; Nedd4$^{fl/fl}$* (*mutants*). Mean +/- SEM; ns, not significant; *^{1}p=0.016, *^{2}p=0.0408, 2 way ANOVA Multiple Comparisons. Source data are provided as a Source Data file.

Supplementary Fig 14

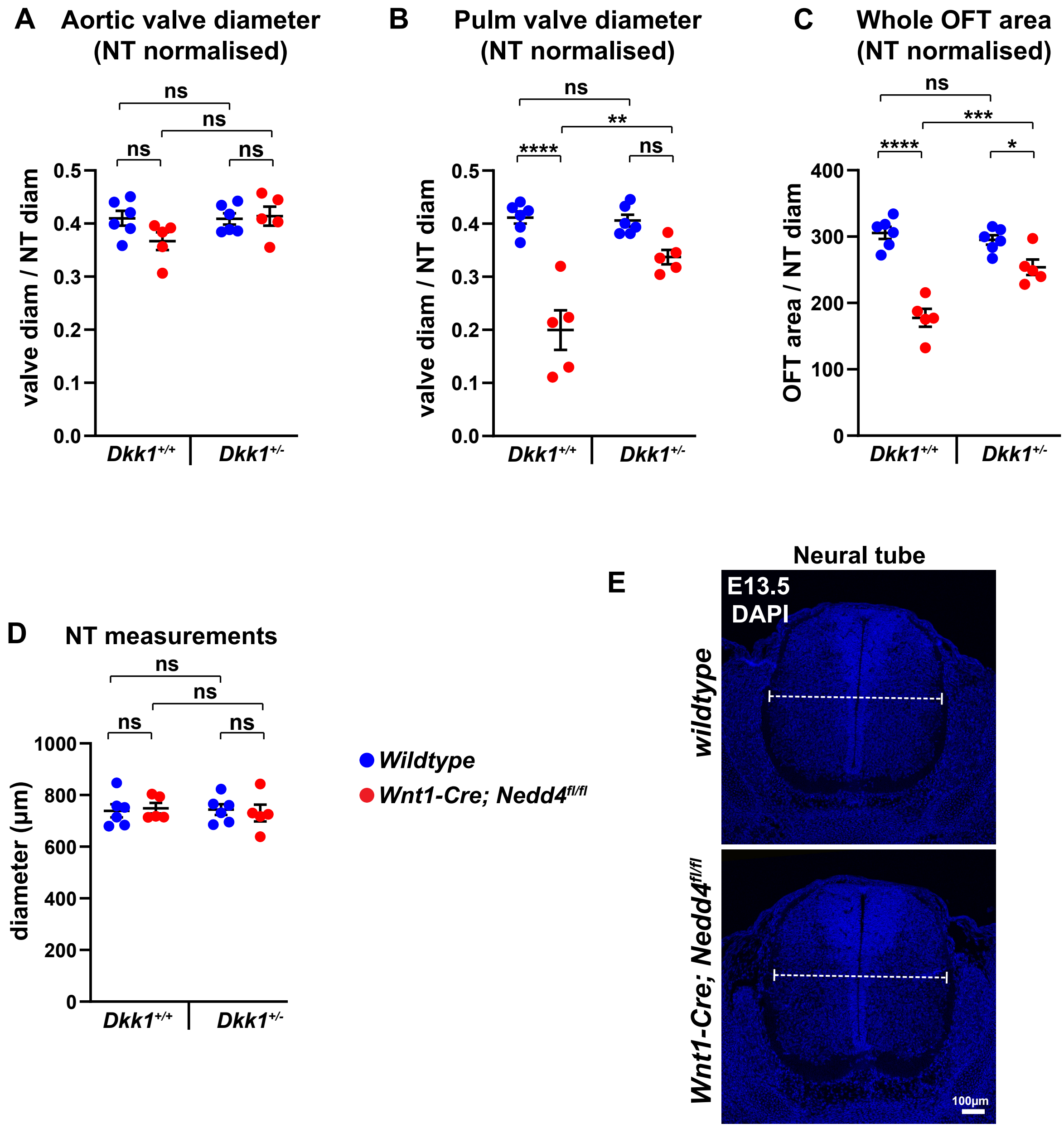

Supplementary Figure 14 - Compound *Wnt1-Cre; Nedd4$^{fl/fl}$*; *Dkk1$^{+/-}$* embryos demonstrate improved outflow tract development.

To account for any variability in embryo size, the valve diameter and outflow tract area measurements in Fig. 5 I, J, K were normalised to the neural tube diameter of the same embryo, which were captured and imaged as part of the same immunostained sections of the outflow tract. 3 neural tube sections were measured per embryo and averaged to use as the normalising factor. **A:** Aortic valve diameter measurements as in Fig. 5I, normalised to neural tube diameter. Mean +/- SEM; ns, not significant. **B:** Pulmonary valve diameter measurements as in Fig. 5J, normalised to neural tube diameter. Mean +/- SEM; ns, not significant; ****p=0.000003; **p=0.001028. 2 way ANOVA multiple comparisons. **C:** Whole outflow tract area measurements as in Fig. 5K, normalised to neural tube diameter. Mean +/- SEM; ns, not significant; ****p=0.0000009; ***p=0.00048; *p=0.0489. 2 way ANOVA multiple comparisons. **D:** Neural tube diameter measurements, demonstrating this structure is unaffected by the genotypes tested. Mean +/- SEM; ns, not significant. **E:** Example image of neural tube diameter measurement (dashed line). Source data are provided as a Source Data file.

Supplementary Figure 15

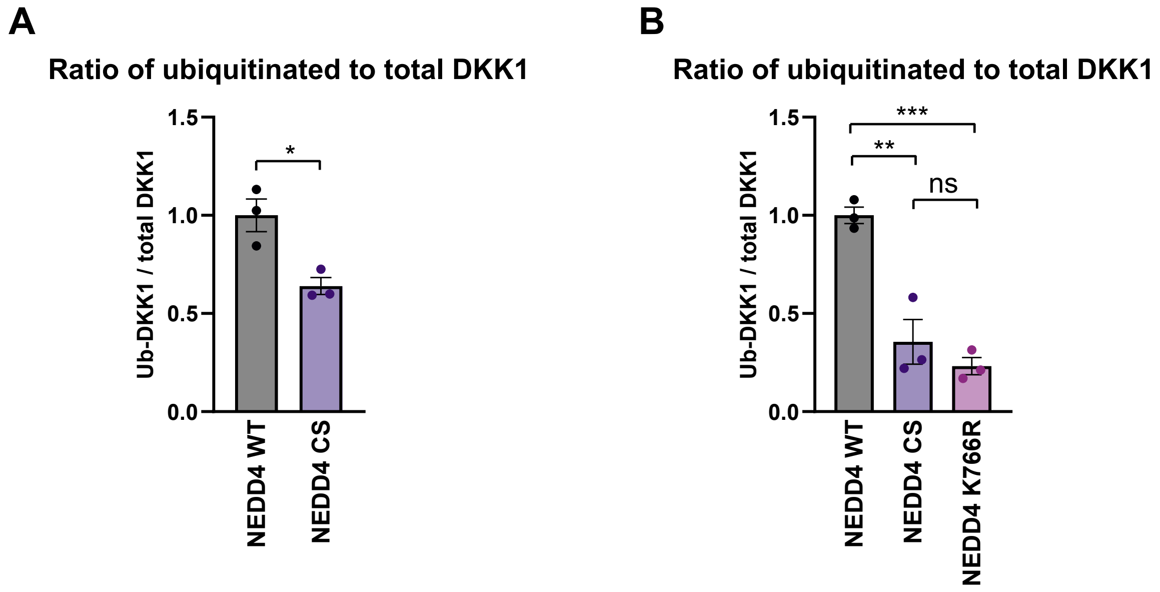

Supplementary Figure 15 – Quantification of ubiquitination assays demonstrates NEDD4 ubiquitinates DKK1, but not a human disease variant NEDD4 K766R.

A: Quantitation of western blots from *in vitro* ubiquitination assay in Figure 6A. Integrated density measurements were taken for the immunoprecipitated (IP) blots for GFP (ie. total DKK1 IP'd) and HA (ie. ubiquitinated portion of DKK1). Ubiquitinated DKK1 is expressed as the ratio of HA density measurement / GFP density measurement. Quantitation was performed for n=3 independent experiments. Mean +/- SEM; * p=0.0186. Unpaired two-tailed t-test. **B:** Quantitation of western blots from *in vitro* ubiquitination assay in Figure 7C. Quantitation was performed for n=3 independent experiments. Measurements performed as in A. Mean +/- SEM; *** p=0.0002; ** p=0.006; ns, not significant. Unpaired two-tailed t-test. Source data are provided as a Source Data file.

Supplementary Figure 16

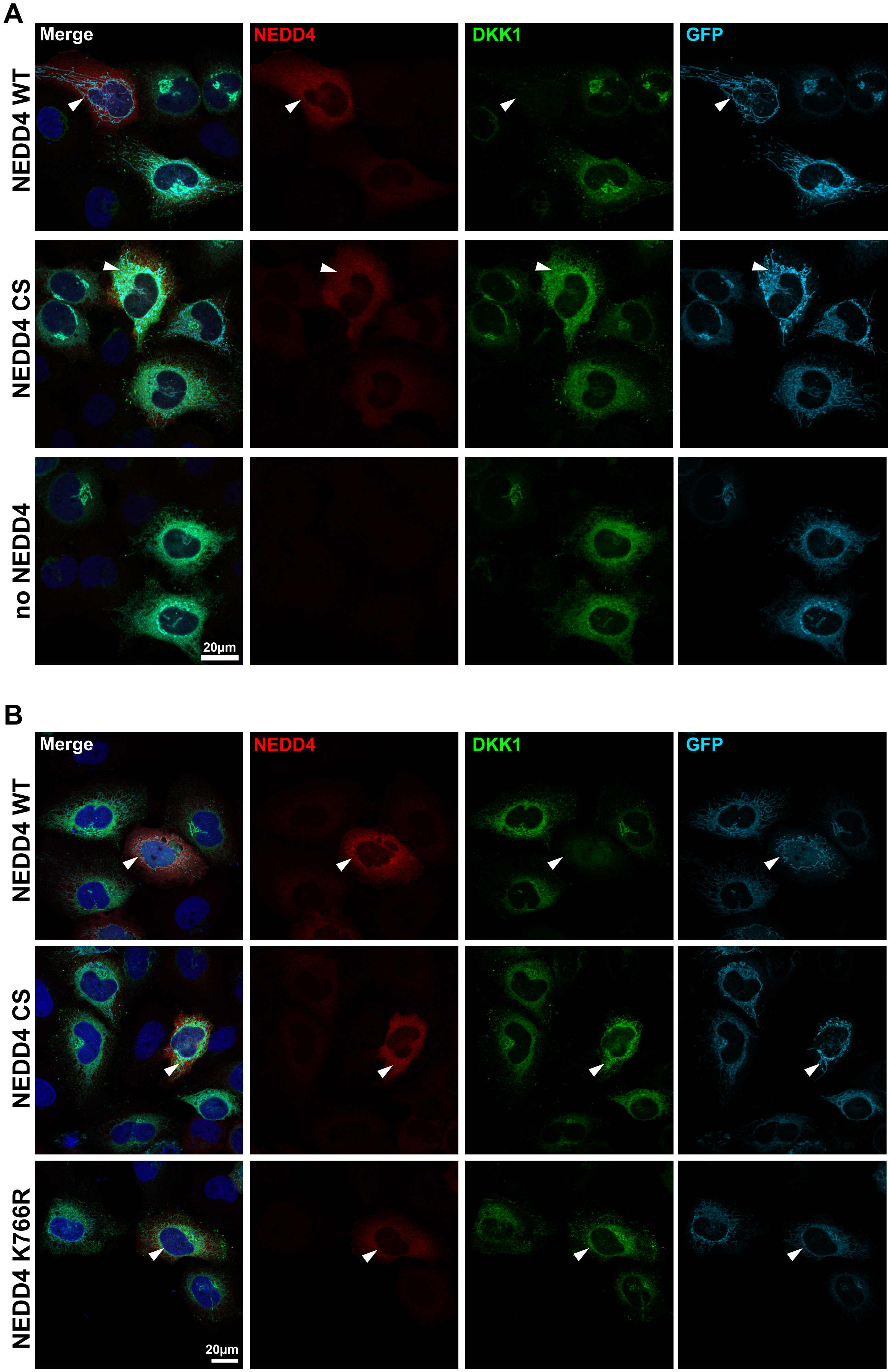

Supplementary Figure 16 – HeLa cell protein abundance correlation assays, additional controls.

A: HeLa cells transfected with DKK1-GFP and FLAG-NEDD4 WT, NEDD4 CS, or no NEDD4 immunostained for FLAG, DKK1 and GFP, as in Figure 6D. GFP immunostaining serves as a transfection control for the DKK1-GFP construct, validating that any reduced DKK1 immunofluorescence in these cells is attributed to post-translational regulation of DKK1 by NEDD4 WT, and not simply due to lack of transfection of the DKK1-GFP construct. No NEDD4 panels serve as an additional control. **B:** Immunostaining of HeLa cells as in A, for Figure 7D, demonstrating GFP immunofluorescence for transfection control.
