# Supplementary material for: Neural crest cell-derived DKK1 and NEDD4 modulate Wnt signalling in the second heart field to orchestrate outflow tract development
Source: Nat Commun. 2026 Jan 22;17:1751. doi: 10.1038/s41467-026-68459-4 (PMC12913648; doi:10.1038/s41467-026-68459-4)
Supplement: Supplementary file 3 — Description of Additional Supplementary Files [file 41467_2026_68459_MOESM3_ESM.pdf]

Description of Additional Supplementary Files

File name: Supplementary Data 1
Description: Laser capture mRNA sequencing data. Excel file containing laser capture RNA sequencing read counts from n=3 *wildtype* (WT) and n=3 *Wnt1-Cre; Nedd4*$^{fl/fl}$ (Mut) embryos. Data includes raw counts, counts per million (CPM), differentially expressed genes (DEGs) calculated from DEGUST analysis, and Wnt regulated genes.
