# Supplementary material for: Neural crest cell-derived DKK1 and NEDD4 modulate Wnt signalling in the second heart field to orchestrate outflow tract development
Source: Nat Commun. 2026 Jan 22;17:1751. doi: 10.1038/s41467-026-68459-4 (PMC12913648; doi:10.1038/s41467-026-68459-4)
Supplement: Supplementary file 5 — Reporting Summary [file 41467_2026_68459_MOESM5_ESM.pdf]

nature portfolio

Corresponding author(s): Sophie Wiszniak
Quenten Schwarz

Last updated by author(s): Dec 18, 2025

Reporting Summary

Nature Portfolio wishes to improve the reproducibility of the work that we publish. This form provides structure for consistency and transparency in reporting. For further information on Nature Portfolio policies, see our Editorial Policies and the Editorial Policy Checklist.

Statistics

For all statistical analyses, confirm that the following items are present in the figure legend, table legend, main text, or Methods section.

n/a	Confirmed	
☐	☒	The exact sample size (*n*) for each experimental group/condition, given as a discrete number and unit of measurement
☐	☒	A statement on whether measurements were taken from distinct samples or whether the same sample was measured repeatedly
☐	☒	The statistical test(s) used AND whether they are one- or two-sided<br>*Only common tests should be described solely by name; describe more complex techniques in the Methods section.*
☒	☐	A description of all covariates tested
☒	☐	A description of any assumptions or corrections, such as tests of normality and adjustment for multiple comparisons
☐	☒	A full description of the statistical parameters including central tendency (e.g. means) or other basic estimates (e.g. regression coefficient) AND variation (e.g. standard deviation) or associated estimates of uncertainty (e.g. confidence intervals)
☐	☒	For null hypothesis testing, the test statistic (e.g. *F*, *t*, *r*) with confidence intervals, effect sizes, degrees of freedom and *P* value noted<br>*Give P values as exact values whenever suitable.*
☒	☐	For Bayesian analysis, information on the choice of priors and Markov chain Monte Carlo settings
☒	☐	For hierarchical and complex designs, identification of the appropriate level for tests and full reporting of outcomes
☒	☐	Estimates of effect sizes (e.g. Cohen's *d*, Pearson's *r*), indicating how they were calculated

Our web collection on statistics for biologists contains articles on many of the points above.

Software and code

Policy information about availability of computer code

Data collection	Histology images (H&E, in situ hybridisation) and whole embryo/heart images were captured using Olympus DP2-AOU1.1 software or Improvision OpenLab 5 software. Confocal immunofluorescense images were captured using ZEN 2.6 (Blue edition) software. Western blot images were captured using BioRad Image Lab Touch Software or FUJIFILM ImageQuant LAS 4000 software.
Data analysis	All graphs and statistical analysis were performed using GraphPad Prism 10. Mean fluorescent intensity, and length/area measurements were made using ZEN 3.4 (Blue edition). Outflow tract rotation measurements were calculated using ImageJ. Densitometry of all western blot data was performed with ImageJ. Optical density measurements of whole embro in situ hybridisation were performed in ImageJ.<br>Laser capture mRNAseq: Raw laser capture mRNAseq data was analysed and quality checked using the FastQC program (http://www.bioinformatics.babraham.ac.uk/projects/fastqc), with duplicate reads removed using Picard Tools (version 2.16.0). Differential expression analysis was evaluated using R (version 3.2.3), edgeR (version 3.3) and Degust software tools. Graphical representations of differentially expressed genes were generated using Glimma 63, Degust and MaGIC Volcano Plot Tool. Gene ontology analysis was performed using DAVID.<br>Calling and annotation of human sequencing data: Sequence reads were mapped to human reference genome hg38 using Burrows-Wheeler Aligner (BWA-mem v0.7.12). Single nucleotide variants (SNVs) and small insertions or deletions were called using Platypus v0.8.1. The variant call files (VCFs) were annotated using ANNOVAR (version 2016Feb01). Variant frequencies were in reference to gnomAD database (v2.1.1).<br>All figures were constructed using Adobe Photoshop 2025 and Adobe Illustrator 2025 software.

For manuscripts utilizing custom algorithms or software that are central to the research but not yet described in published literature, software must be made available to editors and reviewers. We strongly encourage code deposition in a community repository (e.g. GitHub). See the Nature Portfolio guidelines for submitting code & software for further information.

Data

Policy information about availability of data

All manuscripts must include a data availability statement. This statement should provide the following information, where applicable:

- Accession codes, unique identifiers, or web links for publicly available datasets
- A description of any restrictions on data availability
- For clinical datasets or third party data, please ensure that the statement adheres to our policy

Laser capture mRNA sequencing data is available publicly from https://www.ncbi.nlm.nih.gov/geo/ under accession number GSE309818.

Research involving human participants, their data, or biological material

Policy information about studies with human participants or human data. See also policy information about sex, gender (identity/presentation), and sexual orientation and race, ethnicity and racism.

Reporting on sex and gender	Sex was reported for the trio (Father, male; Mother, female; Proband, male) that was subjected to whole genome sequencing.
Reporting on race, ethnicity, or other socially relevant groupings	Data on race or ethnicity was not collected or reported.
Population characteristics	Whole genome sequencing data was obtained for all individuals, and relevant medical history obtained.
Recruitment	The family was recruited through the Kids Heart Research DNA Bank at Children's Hospital at Westmead, Sydney, Australia. Informed, written consent was obtained from the family. Heart defects in the proband was confirmed by echocardiography.
Ethics oversight	Ethics approval was obtained from Sydney Children's Hospital Network Human Research Ethics Committee (approval number HREC/16/SCHN/73).

Note that full information on the approval of the study protocol must also be provided in the manuscript.

Field-specific reporting

Please select the one below that is the best fit for your research. If you are not sure, read the appropriate sections before making your selection.

☒ Life sciences ☐ Behavioural & social sciences ☐ Ecological, evolutionary & environmental sciences

For a reference copy of the document with all sections, see nature.com/documents/nr-reporting-summary-flat.pdf

Life sciences study design

All studies must disclose on these points even when the disclosure is negative.

Sample size	No statistical method was used to calculate sample size. Sample size was chosen empirically according to the variability observed during morphological characterization of the different WT and mutant phenotypes for each of the analyses.
Data exclusions	No data were excluded from the analyses.
Replication	All experimental findings were reliably reproduced. The number of times the experiment was independently replicated is reported in the figure legends where appropriate.
Randomization	Randomisation was not performed, groups were allocated based on embryonic genotype for all analyses performed.
Blinding	Blinding was not performed, as embryonic genotype or cell treatment was known to the investigators for all analyses.

Reporting for specific materials, systems and methods

We require information from authors about some types of materials, experimental systems and methods used in many studies. Here, indicate whether each material, system or method listed is relevant to your study. If you are not sure if a list item applies to your research, read the appropriate section before selecting a response.

Materials & experimental systems

n/a	Involved in the study
	[x] Antibodies
	[x] Eukaryotic cell lines
[x]	Palaeontology and archaeology
	[x] Animals and other organisms
[x]	Clinical data
[x]	Dual use research of concern
[x]	Plants

Methods

n/a	Involved in the study
[x]	ChIP-seq
[x]	Flow cytometry
[x]	MRI-based neuroimaging

Antibodies

Antibodies used	Antibodies used were: rabbit anti-NEDD4 (Abcam 14592) 1:300; rabbit anti-NEDD4 (purified serum, gift from S. Kumar) 1:300; mouse anti-alpha smooth muscle actin (Sigma A2547) 1:2000; rat anti-CD31 (Biolegend 102502) 1:150; goat anti-SOX10 (R&D Systems AF2864) 1:200; chicken anti-GFP (Abcam ab13970) 1:1000; mouse anti-Isl1 (DSHB 39.4D5 or 40.3A4) 1:50; mouse anti-MF20 (DSHB) 1:100; goat anti-DKK1 (R&D Systems AF1765) 1:200; mouse anti-AP2a (DSHB 3B5) 1:20; rabbit anti-beta-catenin non-pS45 (Cell Signaling Technology 19807) 1:200; mouse anti-beta-catenin ABC (8E7) (Millipore 05-665) 1:100; mouse anti-beta-catenin pY489 (DSHB) 1:50; mouse anti-FLAG (Sigma F3165) 1:1000; mouse anti-GM130 (BD 610822) 1:100; rabbit anti-phospho-Histone H3 (Millipore 06-570) 1:500; rabbit anti-cleaved-Caspase-3 (Cell Signaling Technology 9661) 1:500; rabbit anti-phospho-SMAD1/5/9 (Cell Signaling Technology 13820) 1:200; rabbit anti-phospho-ERK1/2 (Cell Signaling Technology 4370) 1:100; goat anti-Scribble (Santa Cruz Biotechnology sc-11048) 1:50; rabbit anti-Laminin (Sigma L9393) 1:1000; rabbit anti-Fibronectin (DakoCytomation A0245) 1:1000; mouse anti-N-cadherin (Cell Signaling Technology 14215) 1:100; mouse anti-HA (6E2, Cell Signaling Technologies 2367) 1:1000; mouse anti-Myc (9B11, Cell Signaling Technologies 2276) 1:1000; rabbit anti-GFP (ClonTech 632592) 1:1000; mouse anti-beta-Actin (Sigma A5441) 1:1000; rabbit anti 14-3-3zeta C-16 (sc1019 Santa Cruz) 1:1000.
Validation	All commercially available antibodies were validated according to tissue expression patterns or molecular weights as per product data sheets, and according to that observed in the literature. Anti-NEDD4 purified serum was validated by absence of immunostaining in conditional NEDD4 knockout mouse tissue.

Eukaryotic cell lines

Policy information about cell lines and Sex and Gender in Research

Cell line source(s)	HEK293T cells were obtained from ATCC (CRL-3216). HeLa cells were kindly provided by the laboratory of Prof. Stuart Pitson (Centre for Cancer Biology, Adelaide), and were originally obtained from ATCC.
Authentication	None of the cell lines were authenticated for this study.
Mycoplasma contamination	All cell lines used have tested negative for mycoplasma contamination.
Commonly misidentified lines (See ICLAC register)	No commonly misidentified cell lines were used in this study.

Animals and other research organisms

Policy information about studies involving animals; ARRIVE guidelines recommended for reporting animal research, and Sex and Gender in Research

Laboratory animals	Species: Mus Musculus<br>Strains used: Nedd4-/-; Nedd4fl/fl; Wnt1-Cre; Tie2-Cre; Mef2cAHF-Cre; Z/EG; Mef2cAHF-LacZ; Dkk1-/-; Nedd4K776R/K766R.<br>All mouse lines were in a mixed C57BL/6 background.<br>Mice were housed with a 12h dark/light cycle, with temperature maintained between 19-23 degrees C and 40-70% relative humidity. Mice used for timed matings were between 6-12 weeks of age.
Wild animals	No wild animals were used.
Reporting on sex	For embryonic analyses we did not genotype for sex, hence this information has not been collected. For laser capture microdissection and RNA sequencing, we aimed to have a balance of sex between the n=3 wildtype and mutant samples (wildtype 2x female, 1x male; and mutant 1x female and 2x male).
Field-collected samples	No field-collected samples were used.
Ethics oversight	All experiments were carried out in accordance with ethical guidelines of and approved by the University of South Australia Animal Ethics Committee.

Note that full information on the approval of the study protocol must also be provided in the manuscript.

Plants

Seed stocks	Not used in this study.
Novel plant genotypes	Not used in this study.
Authentication	Not used in this study.
